# Supplementary material for: Evidence supported by Mendelian randomization: impact on inflammatory factors in knee osteoarthritis
Source: Front Med (Lausanne). 2024 May 28;11:1382836. doi: 10.3389/fmed.2024.1382836 (PMC11165061; doi:10.3389/fmed.2024.1382836)
Supplement: Supplementary file 1 [file Data_Sheet_1.ZIP › data/GCST90274840_5e6/840.forest.pdf]

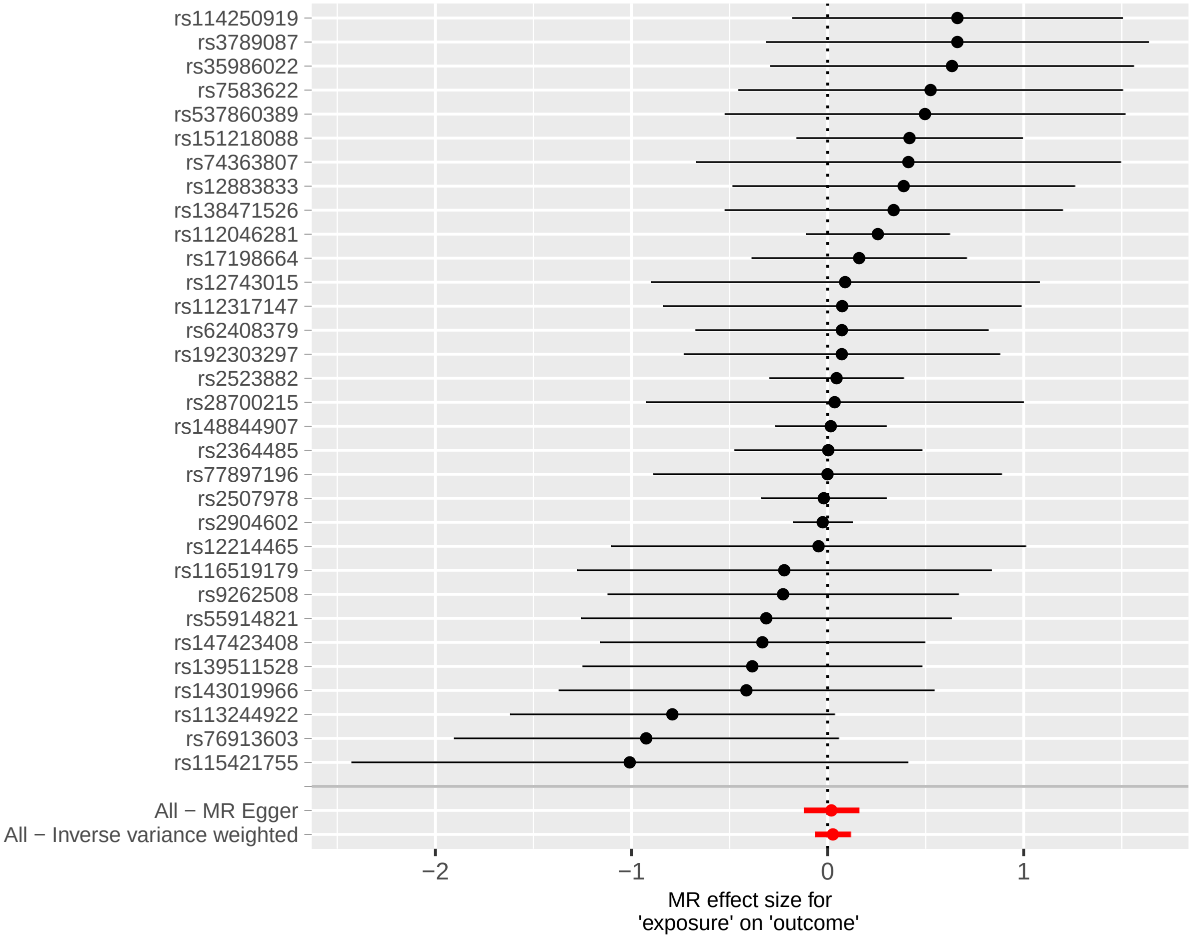
rs114250919
rs3789087
rs35986022
rs7583622
rs537860389
rs151218088
rs74363807
rs12883833
rs138471526
rs112046281
rs17198664
rs12743015
rs112317147
rs62408379
rs192303297
rs2523882
rs28700215
rs148844907
rs2364485
rs77897196
rs2507978
rs2904602
rs12214465
rs116519179
rs9262508
rs55914821
rs147423408
rs139511528
rs143019966
rs113244922
rs76913603
rs115421755
All − MR Egger
All − Inverse variance weighted
−2
−1
0
1
MR effect size for
'exposure' on 'outcome'
